# CSN6-COP1 axis in cancer

**DOI:** 10.18632/aging.100778

**Published:** 2015-07-08

**Authors:** Hyun Ho Choi, Mong-Hong Lee

**Affiliations:** Department of Molecular and Cellular Oncology, The University of Texas MD Anderson Cancer Center, Houston, TX 77030, USA

The association of COP9 signalosome (CSN) with Constitutive Photomorphogenic 1 (COP1) was characterized in plants for a role in photomorpho-genesis. However, since mammalian cells do not perform photomorphogenesis, roles of these two proteins remain enigmatic. Recently we began to unravel their roles in oncogenesis.

The CSN is an evolutionarily conserved multiprotein complex. Mammalian CSN consists of eight subunits (CSN1-CSN8) and has diverse functions, including cell cycle control, signal transduction, and tumorigenesis. Interestingly, CSN6, which is involved in stabilizing MDM2 [1] and preserving Cullin neddylation [2], is overexpressed in many types of cancers, implying its oncogenic role in cancer. However, the mechanistic regulation and biological consequence of CSN6 overexpression in cancer remain to be examined. Mammalian COP1 is an evolutionarily conserved E3 ubiquitin ligase, which contains RING-finger, coiled-coil and WD40-repeat domains. Through association with the CSN, COP1 functions as a crucial mediator to block photomorphogenesis in the dark through regulating the ubiquitinated proteasomal degradation of light-induced transcription factor HY5. In mammalian cells, COP1 is overexpressed in a various types of cancers, and it causes ubiquitin-mediated degradation of tumor suppressor p53, suggesting its oncogenic role in cancer. But its other substrates through protein degradation may have roles in tumorigenesis.

Recently, CSN6's interaction with COP1 is characterized in mammalian cells, and CSN6-COP1 axis is involved in 14-3-3σ degradation [3]. COP1 is an E3 ligase of 14-3-3σ. 14-3-3σ, a tumor suppressor involved in opposing cancer metabolic reprogramming [4] and Akt signaling, is known to be upregulated by p53 and has a positive feedback effect on p53 in response to DNA damage. CSN6 stabilizes COP1 through reducing COP1 self-ubiquitination and thus decelerates COP1's turnover rate. CSN6/COP1-mediated 14-3-3σ ubiquitination is compromised when COP1 is knocked down. Subsequently, CSN6-COP1 axis causes 14-3-3σ downregulation, thereby activating Akt and promoting cancer cell survival.

Also, the CSN6-COP1 axis regulates the stability p27^Kip1^, a critical G1 CDK inhibitor involved in cell cycle brake. COP1 is an E3 ligase of p27 to enhance p27 ubiquitination and subsequent degradation, which acts independent of negative regulators (E3 ligases) of p27, such as SKP2, Jab1, Pirh2, or KPC1 [5, 6]. Ectopic expression of CSN6 decreases the expression of p27 while CSN6 knockdown leads to p27 stabilization. Mechanistic studies show that CSN6 interacts with p27 and facilitates ubiquitin-mediated degradation of p27. CSN6-mediated p27 degradation depends on the nuclear export of p27, which is regulated through COP1's nuclear exporting signal. COP1 overexpression leads to the cytoplasmic distribution of p27, thereby accelerating p27 degradation. Importantly, the negative impact of COP1 on p27 stability contributes to elevated expression of genes that are suppressed through p27 mediation. Also, COP1-mediated p27 translocation to the cytoplasm will promote cancer cell migration and invasiveness, phenomena imposed by p27 cytoplasmic distribution. Kaplan-Meier analysis of tumor samples demonstrates that high COP1 or CSN6 expression is correlated with poor overall survival. These data suggest that tumors with CSN6-COP1 axis activation may have growth advantage by regulating p27 degradation and by subsequently impacting on p27 targeted gene expression.

Understanding the genome integrity and DNA damage response is critical to cancer treatment. CSN6-COP1 axis can regulate genome integrity [7]. CSN6 overexpression leads to mitotic defect and ROS production. Importantly, COP1 and Aurora A (a p27-mediated suppressed gene involved in mitosis) are involved in this process. p27 levels are elevated after DNA damage, with concurrent reduction of COP1 levels. Mechanistic studies demonstrate that during DNA damage response COP1's function as an E3 ligase of p27 is inactivated due to 14-3-3σ-enhanced COP1 degradation, thereby reducing the ubiquitin-mediated degradation of p27. This process of p27 accumulation in response to DNA damage is not p53-dependent, but it is ATM- and 14-3-3σ-dependent. 14-3-3σ collaborates with ATM activity to mediate downregulation of COP1 after DNA damage. Congruently, in 14-3-3σ-null cells, COP1 is not downregulated by DNA damage; therefore, p27 levels are not increased. Expression of 14-3-3σ facilitates the downregulation of COP1 and subsequent increase of p27 in response to DNA damage. Studies show that p27 levels accumulate in the nucleus when COP1 is excluded from the cytoplasm in the presence of the DNA-damaging agent doxorubicin, suggesting that DNA damage affects the subcellular distribution of COP1, which in turn affects p27 levels. Further, COP1 overexpression leads to downregulation of p27^Kip1^, thereby promoting the overexpression of Aurora A, which correlates with poor survival. These findings provide new insight into CSN6-COP1-p27-Aurora A axis in DNA damage repair and tumorigenesis.

Taken together, data show that COP1 is critical in CSN6-mediated oncogenesis, conferring impacts on 14-3-3σ, p27, Aurora A, Akt, metabolic reprogramming, genome integrity, and others. Further studies should be explored to address the following questions. What are other downstream ubiquitination targets of CSN6-COP1 axis involved in cancer? What are the upstream oncogenic signals in regulating this axis to promote cancer growth? Strategy in hindering the CSN6-COP1 axis activation may be a useful therapeutic strategy for cancer intervention.
